# Mastoid notch as a landmark for localization of the transverse-sigmoid sinus junction

**DOI:** 10.1186/s12883-020-01688-2

**Published:** 2020-03-27

**Authors:** Ruichun Li, Lei Qi, Xiao Yu, Kuo Li, Gang Bao

**Affiliations:** Department of Neurosurgery, First Affiliated Hospital of Xi’an Jiaotong University, 277 West Yanta Road, Xi’an, 710061 Shaanxi China

**Keywords:** Transverse sinus, Sigmoid sinus, Mastoid notch, Skull base

## Abstract

**Background:**

The top of the mastoid notch (TMN) is close to the transverse-sigmoid sinus junction. The spatial position relationship between the TMN and the key points (the anterosuperior and inferomedial points of the transverse-sigmoid sinus junction, ASTS and IMTS) can be used as a novel method to precisely locate the sinus junction during lateral skull base craniotomy.

**Methods:**

Forty-three dried adult skull samples (21 from males and 22 from females) were included in the study. A rectangular coordinate system on the lateral surface of the skull was defined to assist the analysis. According to sex and skull side, the data were divided into 4 groups: male&left, male&right, female&left and female&right. The distances from the ASTS and IMTS to the TMN were evaluated on the X-axis and Y-axis, symbolized as ASTS&TMN_x, ASTS&TMN_y, IMTS&TMN_x and IMTS&TMN_y.

**Results:**

Among the four groups, there was no significant difference in ASTS&TMN_x (*p* = 0.05) and ASTS&TMN_y (*p* = 0.3059), but there were significant differences in IMTS&TMN_x (*p* < 0.001) and IMTS&TMN_y (*p* = 0.01), and multiple comparisons indicated that there were significant differences between male&left and female&left both in IMTS&TMN_x (*p* = 0.0006) and in IMTS&TMN_y (*p* = 0.0081). In general, the ASTS was located 1.92 mm anterior to the TMN on the X-axis and 27.01 mm superior to the TMN on the Y-axis. For the male skulls, the IMTS was located 3.60 mm posterior to the TMN on the X-axis and 14.40 mm superior to the TMN on the Y-axis; for the female skulls, the IMTS was located 7.84 mm posterior to the TMN on the X-axis and 19.70 mm superior to the TMN on the Y-axis.

**Conclusions:**

The TMN is a useful landmark for accurately locating the ASTS and IMTS.

## Background

The anterosuperior and inferomedial points of the transverse-sigmoid sinus junction (ASTS and IMTS) represent the most posterior edge of the middle fossa and the most superolateral limit of the retrosigmoid approach, respectively. Accurately locating these key points on the external surface of the cranium is important in lateral skull base craniectomy [1–4]. Traditionally the squamosal-parietomastoid suture junction (SP) and asterion have been regarded as the classic landmarks for assisting with identifying the ASTS and IMTS, respectively [5–7]. However, the cranial sutures is covered by periosteum, and this condition often leads to the inability to recongnize the SP and asterion [7, 8]. Therefore, it is necessary to find a more reliable and practical method to precisely locate the key points.

The mastoid notch is a deep groove on the medial side of the mastoid process that can be clearly identified. Because the top of the mastoid notch (TMN) is close to the transverse-sigmoid sinus junction, it is possible to make it a potential landmark for locating the ASTS and IMTS [9, 10].

The purpose of this study was to analyze the spatial position relationship between the TMN and the ASTS and IMTS and to provide a new method for the accurate positioning of these key points in lateral skull base craniectomy.

## Methods

This study was approved by the Ethics Committee of the First Affiliated Hospital of the Medical College of Xi’an Jiaotong University (KYLLSL-2014-129-01).

### Skull samples

Forty-three dried adult skulls (age ≥ 18 years, 86 sides) were provided by the Department of Anatomy at the Medical College of Xi’an Jiaotong University. The bony sulci of the transverse and sigmoid sinuses as well as the mastoid notch were clearly discernible. Of the skulls, 21 were from males, and 22 were from females. The skull circumferences were measured through intercilium anteriorly and the external occipital protuberance posteriorly.

### Definations of the ASTS, IMTS and TMN

**Fig. 1:**
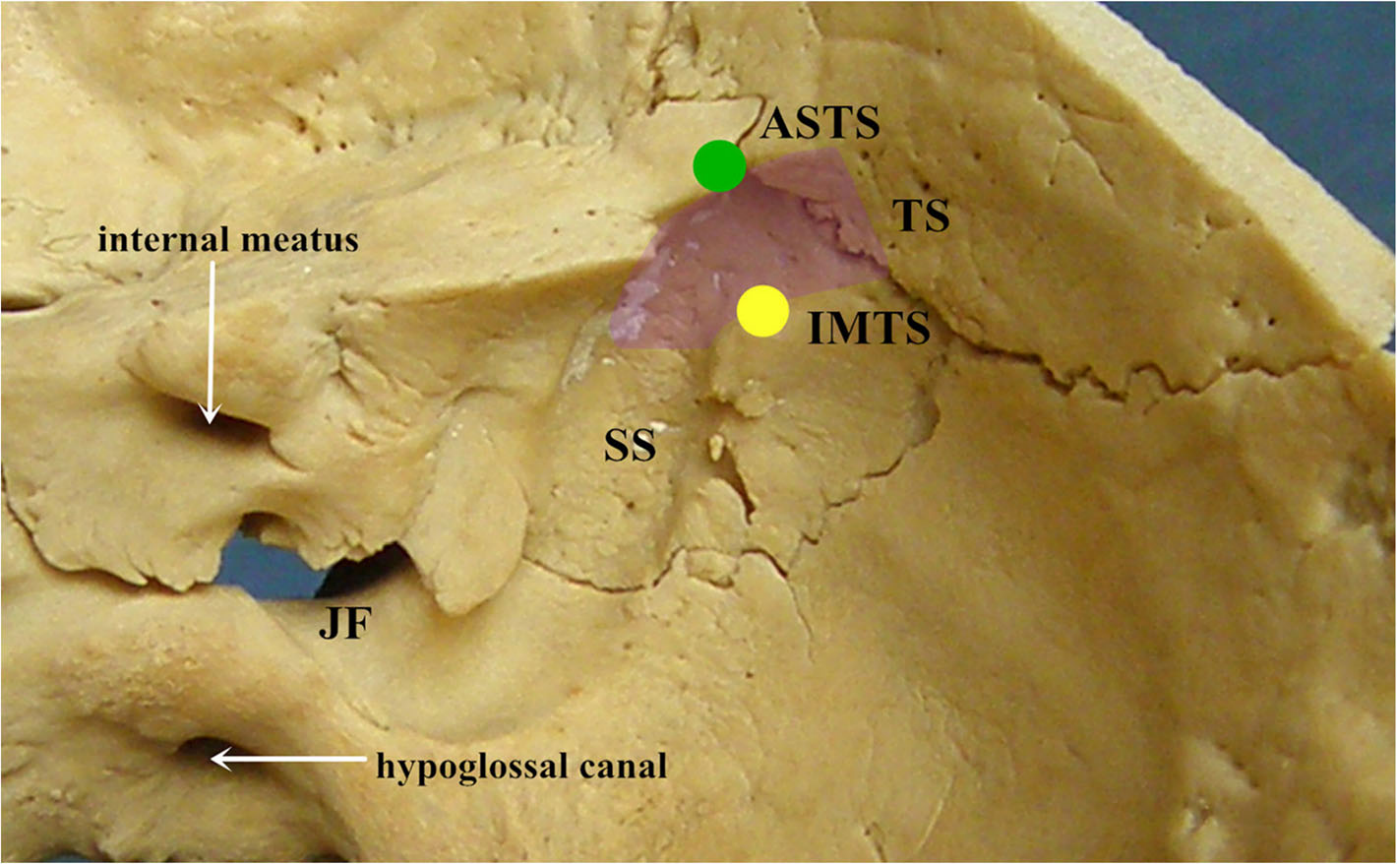
Inner surface of the cranium. The green and yellow circles represent the sites of the ASTS and IMTS, respectively. The purple translucent area represents the transverse-sigmoid sinus junction. TS: transverse sinus; SS: sigmoid sinus; JF: jugular foramen

**Fig. 2:**
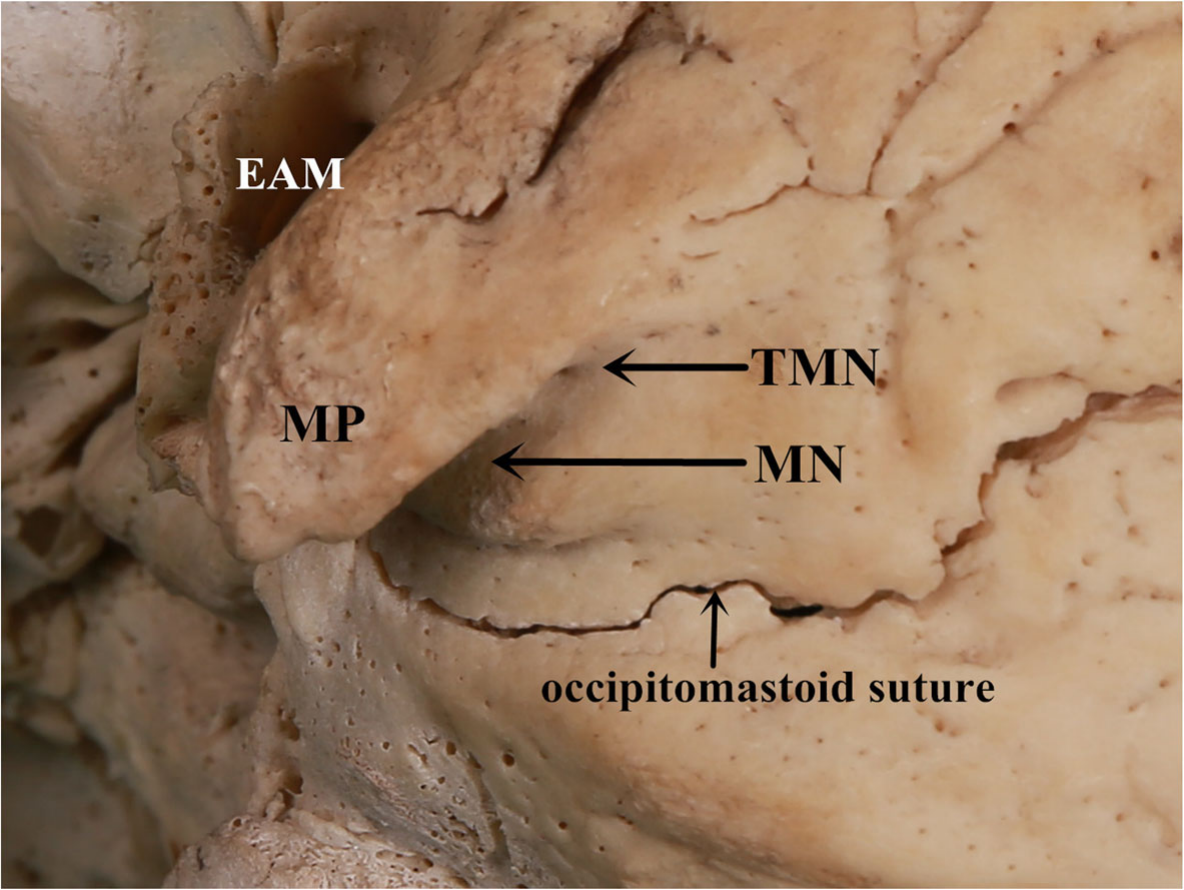
Outer surface of the cranial base. On the medial side of the mastoid process, the mastoid notch extends superomedially to end up as the top of the notch. MP: mastoid process; MN: mastoid notch; TMN: top of the mastoid notch; EAM: external auditory meatus

The transverse-sigmoid sinus junction was defined as the transitional zone where the transverse sinus ended by the vertical descending segment of the sigmoid sinus; the ASTS and IMTS were identified as the anterosuperior and inferomedial points of the junction, respectively [8, 11] (Fig. 1). The projection of the two key points to the outer surface of the skulls was marked with a colored pencil. The TMN was defined as the most superomedial point of the notch (Fig. 2).

### Reference coordinate system

**Fig. 3:**
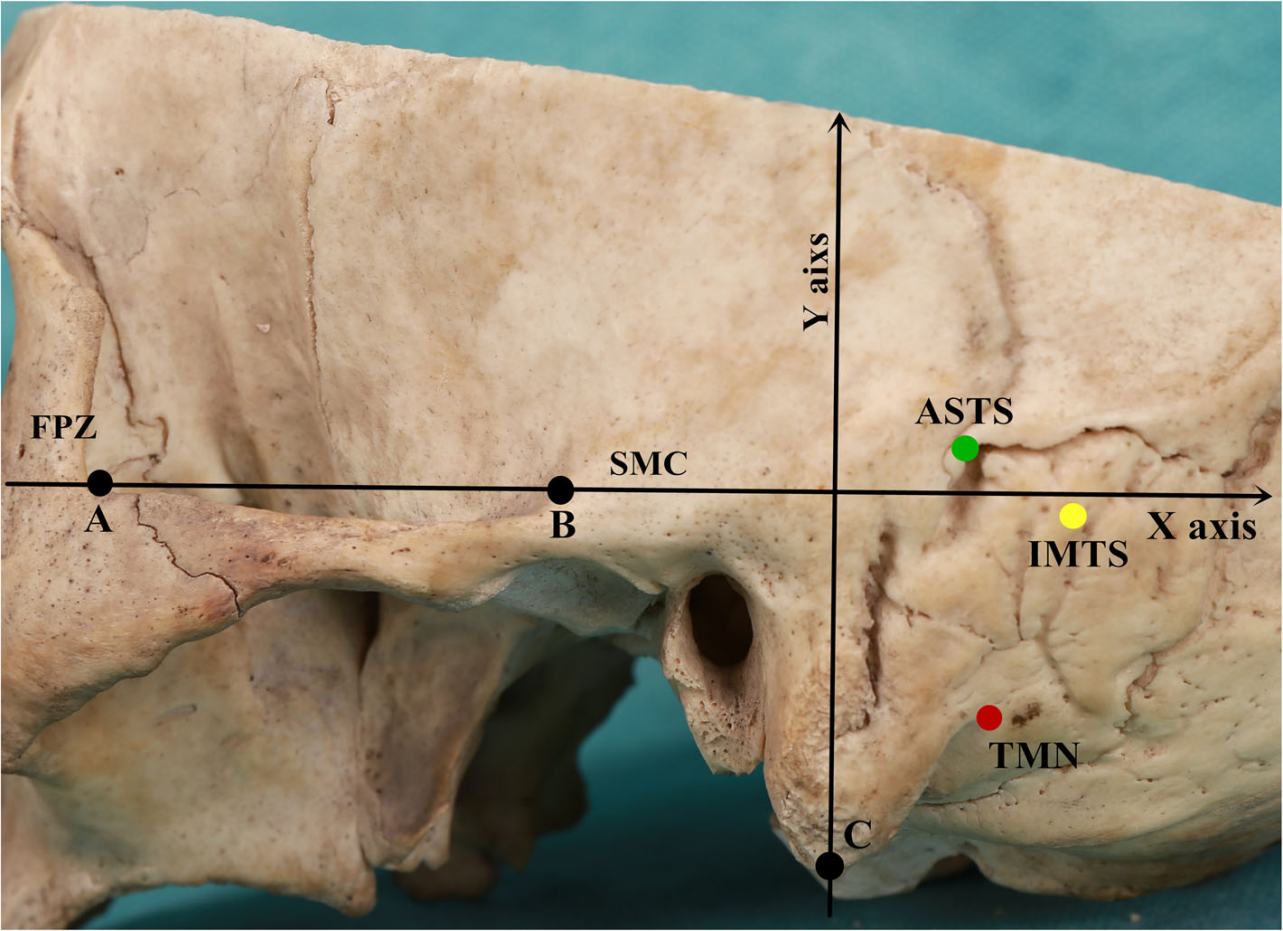
Illustration of the coordinate system on the left side of a skull sample. The X-axis is established by the horizontal line connecting points A and B, which are located where the upper edge of the ZA joins anteriorly to the FPZ and blends posteriorly into the SMC, respectively. The Y-axis is defined by a line through the tip of the mastoid (Point C) and perpendicular to the X-axis. The red, yellow and green circles represent the TMN, IMTS and ASTS, respectively. FPZ: frontal process of the zygomatic bone; ZA: zygomatic arch; SMC: supramastoid crest

A reference rectangular coordinate system was devised to help analyze the spatial position relationship between the TMN and the ASTS and IMTS on the external surface of the skull. The X-axis was defined by points A and B. Point A was located where the upper edge of the zygomatic arch (UEZA) joins anteriorly with the frontal process of the zygomatic bone (FPZ), and point B was located where the UEZA blends posteriorly into the supramastoid crest (SMC). The Y-axis was a straight line that passed through the tip of the mastoid (point C) and was perpendicular to the X-axis. On the X-axis, the posterior side was positive, and the anterior side was negative. On the Y-axis, the superior side was positive, and the inferior side was negative (Fig. 3).

The ASTS, IMTS and TMN were vertically projected onto the axes. ASTS_x, IMTS_x and TMN_x represent the coordinates of the ASTS, IMTS and TMN on the X-axis, respectively; ASTS_y, IMTS_y and TMN_y represent the coordinates of those points on the Y-axis, respectively.

**Fig. 4:**
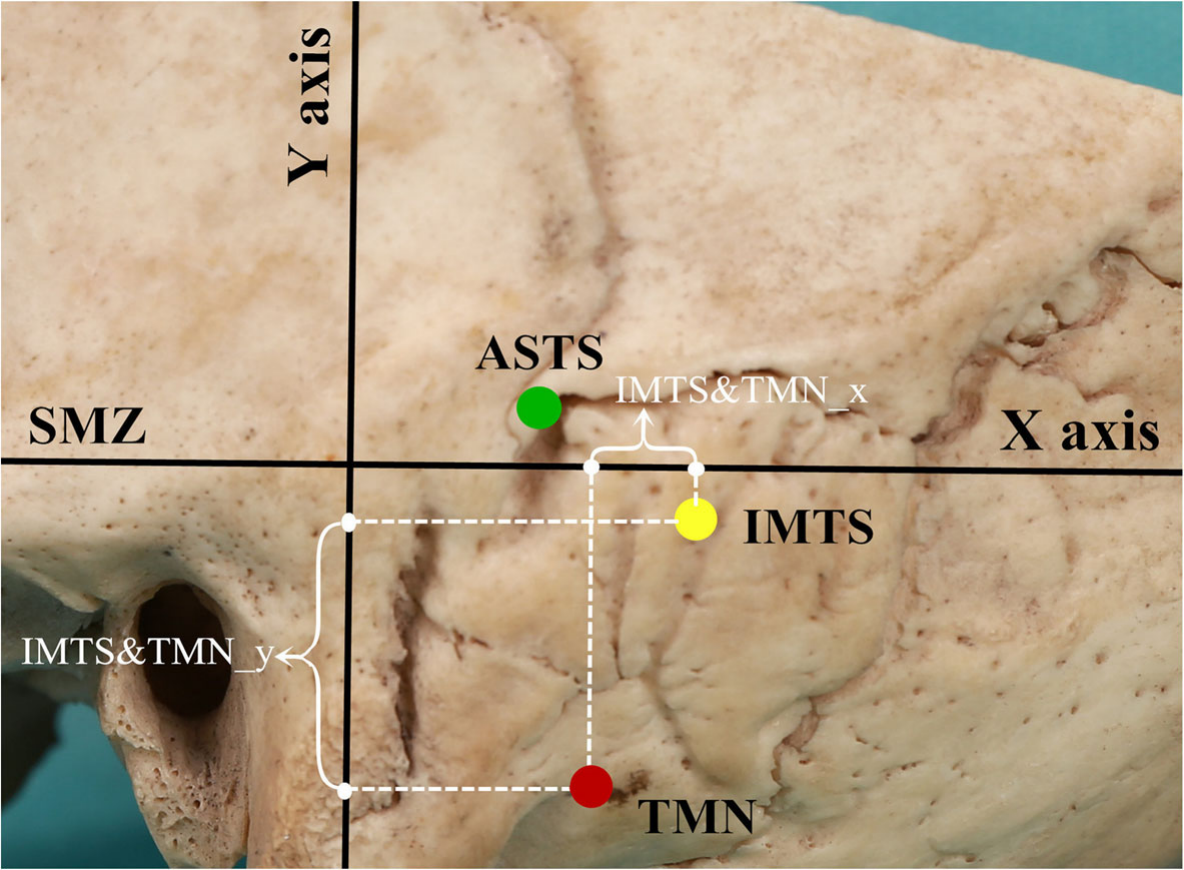
The distances from the IMTS to the TMN were analyzed in the X- and Y-axes directions, symbolized as IMTS&TMN_x and IMTS&TMN_y, respectively. So did that from the ASTS to the TMN

The distances from the ASTS to the TMN were calculated on the X-axis and Y-axis separately and were denoted by ASTS&TMN_x and ASTS&TMN_y (ASTS&TMN_x = ASTS_x – TMN_x, ASTS&TMN_y = ASTS_y – TMN_y). Similarly, the distances from the IMTS to the TMN were denoted by IMTS&TMN_x and IMTS&TMN_y (IMTS&TMN_x = IMTS_x – TMN_x, IMTS&TMN_y = IMTS_y – TMN_y) (Fig. 4).

### Statistical analyses

Two researchers in this study measured the values of the ASTS, IMTS and TMN in the coordinate system using a Vernier caliper with anaccuracy of 0.02 mm. Each sample was measured three times, and the average value was taken as the result. According to sex and skull side, the data were divided into 4 groups: male&left, male&right, female&left and female&right. A normality test was performed using the Shapiro-Wilk test (*α* = 0.1), and the homogeneity of variance test (*α* = 0.1) was conducted by the Levene test. When the variables conformed to a normal distribution, the mean ± standard deviation was used for statistical description; otherwise, the median was used.

To estimate the group size, a pilot study was conducted to measure ASTS&TMN_x, ASTS&TMN_y, IMTS&TMN_x, and IMTS&TMN_y in 20 skulls. With a two-tailed α = 0.05 and a power of 80%, the study required 31 skulls. Ultimately, 43 skull specimens were measured in this study.

Because not all the variables conformed to the normal distribution, Dunn’s test with Bonferroni adjustment of the *p*-value was used for multigroup rank sum test. When the differences in the average levels among groups were statistically significant, the multiple comparisons were carried out. A two-tailed *p* < 0.05 indicated statistical significance. All statistics were performed with R version 3.5.0 (Copyright (C) 2018 The R Foundation for Statistical Computing).

## Results

### Skull circumference and key point coordinates

The average male and female skull circumferences were 49.88 ± 1.19 cm and 48.93 ± 1.13 cm, respectively. The medians of the ASTS, IMTS and TMN coordinates are presented in Table 1.

**Table 1:** Coordinates of the ASTS, IMTS and TMN

	ASTS_x	ASTS_y	IMTS_x	IMTS_y	TMN_x	TMN_y
median, mm						
ML	16.48	7.45	20.78	−4.26	18.36	−18.36
MR	16.32	8.01	25.18	−4.78	19.76	− 19.84
FL	15.23	7.26	21.77	−1.60	16.00	−20.60
FR	15.41	7.44	24.20	−4.43	16.71	−21.00

*M* male, *F* female, *L* left side, *R* right side

The relative coordinates between the ASTS and IMTS and the TMN are shown in Table 2.

**Table 2:** Relative coordinates between key points and TMN

	ASTS&TMN_x	ASTS&TMN_y	IMTS&TMN_x	IMTS&TMN_y
median, mm				
ML	−2.84	26.23	2.56	14.32
MR	−3.16	27.47	4.44	14.48
FL	0.19	27.66	8.38	18.69
FR	−1.40	27.15	7.57	19.74
overall	−1.92	27.01	5.46	15.95

*M* male, *F* female, *L* left side, *R* right side. For the x relative coordinates, sign "- "means the key point was located on the frontal side of the TMN; For the y relative coordinates, −"means the key point was located on the inferior side of the TMN

### ASTS and TMN

The ASTS was located 1.92 mm anterior to the TMN on the X-axis and 27.01 mm superior to the TMN on the Y-axis (see Table 2 for details). There were no significant differences among the 4 groups in ASTS&TMN_x (*p* = 0.05) or ASTS&TMN_y (*p* = 0.3059) (see Table 3 for details).

**Table 3:** Comparisons between genders and sides for ASTS&TMN and IMTS&TMN

		ASTS&TMN_x (*p* = 0.05)	ASTS&TMN_y (*p* = 0.3059)	IMTS&TMN_x (*p* < 0.001)	IMTS&TMN_y (*p* = 0.01)
ML vs MR	*p*=	1.000	0.4821	0.1581	1.000
FL vs FR		0.5404	1.000	1.000	1.000
ML vs FL		0.1340	0.2559	0.0006*	0.0081*
MR vs FR		0.5173	1.000	0.075	0.5603

Dunn test with Bonferroni adjustment of the *p*-value was used for multi-group rank sum test; * *p < 0.05*

### IMTS and TMN

There were significant differences among the 4 groups in IMTS&TMN_x (*p* < 0.001) and IMTS&TMN_y (*p* = 0.01) (see Table 3 for details). Furthermore, multiple comparisons indicated that there were significant differences between male&left and female&left both in IMTS&TMN_x (2.56 mm vs 8.38 mm, *p* = 0.0006) and in IMTS&TMN_y (14.32 mm vs 18.69 mm, *p* = 0.0081). Then, the medians of the IMTS&TMN_x and IMTS&TMN_y were calculated according to sex (see Table 4 for details). For the male skulls, the IMTS was 3.60 mm posterior to the TMN on the X-axis and 14.40 mm superior to the TMN on the Y-axis; for the female skulls, the IMTS was 7.84 mm posterior to the TMN on the X-axis and 19.70 mm superior to the TMN on the Y-axis.

**Table 4:** IMTS&TMN_x and IMTS&TMN_y of the male and female

	IMTS&TMN_x	IMTS&TMN_y
median, mm		
Male	3.60	14.40
Female	7.84	19.70

## Discussion

Over the past decades, neurosurgeons have been using innovative techniques to safely expose the transverse and sigmoid sinuses without resulting in extensive bony defects during craniotomy. The accurate placement of the keyhole at the transverse-sigmoid sinus junction is one of the most important steps in this procedure [11–14]. Goto T used the lateral end of the transverse sinus, the ASTS, the mastoid emissary foramen and the midpoint of the transverse sinus as the site of 4 key holes to complete the exposure of the sigmoid sinus [2]. To avoid extensive bony defects in the periauricular area, Jia recommended a two-bone flap craniotomy technique for the transpetrosal presigmoid approach; this technique required that the first bone flap distinctly expose the ASTS to facilitate the dissection of the sigmoid sinus away from the inner table of the mastoid bone [15]. Usually, the squamosal-parietomastoid suture junction (SP) and the intersection of the supramastoid crest with the squamosal suture (SCSS) are accepted as important landmarks for representing the ASTS [5, 7, 15, 16]. Unfortunately, the squamosal and parietomastoid sutures are often difficult to recognize during craniotomy, especially in older adults. Therefore, the SP and the intersection of the SCSS are not reliable landmarks for the ASTS [8, 17].

The asterion is located at the junction of the parietomastoid, lambdoid and occipitomastoid sutures. It was once considered a classic bony landmark for the IMTS [18, 19]. However, there is growing evidence that placing key holes in the asterion may cause unexpected sinus damage [8, 20–22]. Teranishi investigated the distance from the transverse-sigmoid sinus junction to the asterion by using three-dimensional computed tomography images, and his study indicated that the keyhole should be placed 6.5 mm both laterally and caudally to the asterion [21]. Moreover, the asterion is often difficult to recognize because it is sometimes difficult to identify the parietomastoid, lambdoid and occipitomastoid sutures. Therefore, the asterion it is not a reliable landmark for locating the IMTS [8, 23, 24].

Image-guided surgical planning, including neuronavigation and other methods based on the 3D volume rendering (3D VR) technique, can yield morphometric data in individual patients and can overcome extreme individual variations [7, 11, 25, 26]. For instance, Xia utilized a line connecting the digastric point and the asterion to establish a coordinates systemon for 3D VR images; then he analyzed the coordinate relationship between the asterion and the IMTS to assist with localizing the IMTS [22]. However, these techniques are both expensive and time-consuming. Moreover, they may be limited by equipment problems, emergency cases and allergies to contrast medium [8, 22].

In summary, the methods mentioned above have the following shortcomings: 1) skull sutures sometimes can not be identified distinctly, which makes the positioning ineffective;, 2) traditional landmarks, such as the asterion, are not precise enough to locate the key point; and 3) image-guided surgical planning is both expensive and time-consuming. Therefore, it is still worth identifying an accurate, fast, practical and low-cost method to locate the sinuses.

Tubbs introduced a reference coordinate system established by the X-axis extending along the most superior border of the zygomatic arch and the Y-axis extending from the mastoid notch to the squamosal suture [8]. In 100 adult skulls (200 sides), the distances from the IMTS to the X- and Y-axes were measured and analyzed statistically, and the results were used to locate the IMTS in the retrosigmoid approach. This method does not require the identification of any skull sutures. Over the past few years, the method has been modified, but in practice, it is difficult to precisely define the X- and Y-axes; slight coordinate translation or coordinate rotation can lead to major errors in the locating system [20, 24].

In the present study, points A, B and C could be easily identified during craniotomy [3, 8, 20, 24]. However, the most important part of this study was that we utilized a reference point, the TMN, to improve the accuracy of localization. Because this method is based on the relative position between the TMN and the ASTS or IMTS, it is not affected by coordinate translation. In other words, the relative coordinates remain constant when the coordinate system is translated. Therefore, this new localization method should be more accurate and practical than before [8, 20, 24].

### Application of this study

Before disinfection of the operation area, a colored pen should be used to outline the X- and Y-axes on the scalp. After exposing the bony surface of the skull base, the axes should be marked on the bone surface by unipolar electrocoagulation, and the TMN point can be found at the same time.

#### ASTS and TMN

Because there was no significant difference in sex or skull side, the ASTS can be located 1.92 mm anterior to and 27.01 mm superior to the TMN with directions parallel to the X- and Y-axes, respectively.

#### IMTS and TMN

There were significant differences between male and female skulls both in IMTS&TMN_x and in IMTS&TMN_y, which indicate that the distance from the IMTS to the TMN in males was shorter than that in females. Then, for the male skulls, the IMTS can be located 3.60 mm posterior to and 14.40 mm superior to the TMN; for the female skulls, the IMTS can be located 7.84 mm posterior to and 19.70 mm superior to the TMN. Additionally, the directions should be parallel to the X- and Y-axes.

### Limitation of the study

Since the data in this study were obtained from 43 adult skulls, the results should not be applied to children. Additionally, there was occasionally a high degree of individual variation in the relationship between the TMN and the key points, which thus increases the risk of sinus injury [25, 26]. Therefore when conditions permit, neuronavigation and other image-assisted positioning technologies should be taken into account to overcome extreme variations.

## Conclusions

The TMN is a useful landmark for accurately locating the ASTS and IMTS. However, The results of this study are only applicable to adult cases.

## Data Availability

All data generated or analysed during this study are included in this published article.

## Abbreviations

ASTS: The anterosuperior point of the transverse-sigmoid sinus junction
FPZ: The frontal process of the zygomatic bone
IMTS: The inferomedial point of the transverse-sigmoid sinus junction
JF: Jugular foramen
MN: Mastoid notch
MP: Mastoid process
SMC: Supramastoid crest
TMN: The top of the mastoid notch
UEZA: Upper edge of the zygomatic arch
